# Foco na Embolia Pulmonar Aguda de Risco Intermediário. A Combinação de Biomarcadores é a Solução?

**DOI:** 10.36660/abc.20240075

**Published:** 2024-04-09

**Authors:** Carlos Henrique Miranda

**Affiliations:** 1 Universidade de São Paulo Faculdade de Medicina de Ribeirão Preto Departamento de Clínica Médica Ribeirão Preto SP Brasil Divisão de Medicina de Emergência, Departamento de Clínica Médica, Faculdade de Medicina de Ribeirão Preto, Universidade de São Paulo, Ribeirão Preto, SP – Brasil

**Keywords:** Embolia Pulmonar, Biomarcadores, Ecocardiografia

A incidência de embolia pulmonar aguda (EPA) vem aumentando ao longo do tempo em todo o mundo, inclusive no Brasil. Este comportamento é provavelmente secundário ao envelhecimento da população associado ao aumento da prevalência e melhor prognóstico relacionado ao câncer.^1^

A apresentação clínica da EPA apresenta dois extremos fáceis de identificar. Grupo de alto risco, cerca de 4% dos pacientes, que se caracteriza por choque circulatório ou parada cardiorrespiratória, com alta taxa de mortalidade. Neste grupo, o tratamento com trombolíticos está bem estabelecido.^2^

No outro extremo, está o grupo de baixo risco, cerca de 40% dos pacientes, que se caracteriza pelo escore no Índice de Gravidade da Embolia Pulmonar (PESI) ≤ 2 e ausência de dilatação do ventrículo direito (VD) avaliada por ecocardiografia ou angiografia pulmonar por tomografia computadorizada (APTC), com taxa de mortalidade reduzida. Neste grupo, os anticoagulantes, como a enoxaparina ou os anticoagulantes orais de ação direta (AOACs), são suficientes. A alta hospitalar precoce ou mesmo o tratamento ambulatorial podem ser considerados para esses pacientes.^2^

Contudo, o maior desafio na prática clínica é o manejo dos pacientes de risco intermediário, que se encontram entre esses dois extremos. Esse perfil corresponde à maioria dos pacientes com EPA, observado em aproximadamente 56% deles. Esse grupo é altamente heterogêneo e um percentual deles apresenta alta probabilidade de deterioração clínica, comportando-se mais próximo de pacientes de alto risco.^3^

Vários biomarcadores e exames de imagem têm sido utilizados para melhorar a estratificação de risco nesse grupo intermediário. A troponina I ou T é o biomarcador mais utilizado para esse fim. A diretriz da Sociedade Europeia de Cardiologia (ESC) de 2019 recomenda o uso de troponina dentro de um algoritmo de tomada de decisão.^4^ Pacientes com elevação desse biomarcador associado à dilatação do VD são reclassificados como risco intermediário-alto. No entanto, esta recomendação foi baseada em opiniões de especialistas. A maioria dos estudos não utilizou dosagens ultrassensíveis de troponina e seu ponto de corte não foi padronizado.^5^

O peptídeo natriurético tipo B (BNP) e a fração N-terminal do pró-BNP (NT-proBNP) também podem ser usados neste cenário. Uma meta-análise de cinco estudos mais recentes mostrou que um NT-proBNP superior a 1000ρg/ml aumentou o risco de deterioração clínica. No entanto, este marcador não foi avaliado dentro de um algoritmo de tomada de decisão.^6^

O lactato plasmático, amplamente utilizado em pacientes com sepse, também apresentou boa acurácia preditiva em pacientes com EPA para determinar o prognóstico à curto prazo. Adicionando a medição de lactato ao algoritmo ESC-2019, os pacientes classificados como de risco intermediário-alto com lactato venoso ≥ 3,3 mmol/L tiveram uma prevalência de eventos adversos de 27,5% em comparação com 6,8% naqueles com lactato < 3,3 mmol/L.^7^

A dilatação do VD na APTC avaliada pela relação entre o diâmetro diastólico do VD e o diâmetro diastólico do ventrículo esquerdo (valor de corte de 0,9 ou 1,0) é um marcador prognóstico, assim como a dilatação do VD, a hipocinesia da parede livre do VD e a presença de hipertensão pulmonar na ecocardiografia.^8^

Dentro deste contexto, Gunes et al.^9^ mostraram em seu estudo que a forma solúvel de um receptor de interleucina-33 denominado sST2 apresentou boa acurácia (79,8%) na previsão da ocorrência de morte em seis meses e de hospitalização recorrente em pacientes com EPA. Esses investigadores incluíram uma amostra de conveniência de 100 pacientes internados no pronto-socorro. Assim como a grande maioria dos outros estudos que avaliaram biomarcadores na EPA, estudaram o desempenho desse biomarcador independentemente da classificação prognóstica inicial desses pacientes. A validação destes biomarcadores precisaria ser realizada dentro de uma amostra selecionada de pacientes de risco intermediário, excluindo pacientes de baixo e alto risco. O maior desafio na prática clínica é definir com precisão o prognóstico em pacientes de risco intermediário.

Apesar de diversas investigações mostrarem que esses marcadores são preditores independentes de piores desfechos clínicos, seu desempenho preditivo de prognóstico isolado foi insatisfatório. Todos esses biomarcadores apresentaram baixo valor preditivo positivo.^6^

**Figura 1:**
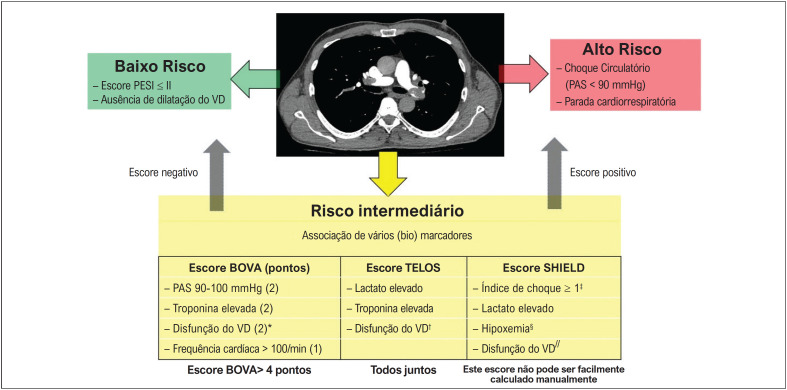
Algoritmo sugerido para estratificação de risco de embolia pulmonar aguda. PESI: índice de gravidade da embolia pulmonar; VD: ventrículo direito; PAS: pressão arterial sistólica; *definido como avaliação ecocardiográfica VD/VE > 0,9, pressão sistólica da artéria pulmonar > 30 mmHg, diâmetro diastólico final do VD > 30mm, dilatação do VD ou hipocinesia de parede livre do VD ou VD/VE > 1,0 na APTC. ^†^ dilatação do VD (diâmetro diastólico final > 30 mm) ou diâmetro diastólico final do VD/VE ≥ 1; hipertensão pulmonar > 30 mmHg, hipocinesia da parede livre do VD; ^‡^frequência cardíaca/pressão arterial sistólica (o valor deve ser inserido em um modelo); ^§^Relação PO_2_/FiO_2_ (o valor deve ser inserido em um modelo); ^//^presença cumulativa de troponina elevada, NT-proBNP elevado e VD/VE ≥ 1,0.

A recomendação atual é utilizar esses biomarcadores dentro de algoritmos preditivos, considerando diversas variáveis em conjunto. Por exemplo, têm surgido escores derivados de estudos prospectivos que associam diversas variáveis, como o escore BOVA.^10^ Na Figura 1 , os pacientes com escore BOVA > 4 pontos apresentaram alta incidência cumulativa de complicações relacionadas ao EPA (19,9%). Outro escore, TELOS, engloba dilatação do VD, troponina e lactato.^11^ Em um estudo clínico, os escores BOVA e TELOS classificaram a mesma proporção de pacientes na categoria de risco intermediário-alto (5,9% e 5,7%) e com taxa de eventos adversos semelhante (18,6% e 21,1%), enquanto o algoritmo ESC 2019 classificou maior percentual de pacientes nesta categoria (12,5%; p<0,001) com menor taxa de eventos (13%; p=0,18).^12^

Até agora, Gunes et al.^9^ completaram a primeira etapa de validação do sST2 na estratificação de risco de EPA. É necessário avaliar o desempenho preditivo deste biomarcador em amostras maiores e multicêntricas, incluindo essencialmente pacientes de risco intermediário, e integrá-lo em algoritmos preditivos com outras variáveis.

A combinação de diversas variáveis, incluindo diferentes biomarcadores, é provavelmente a estratégia mais apropriada para melhorar a estratificação prognóstica na EPA de risco intermediário.
