# Supplementary material for: Foco na Embolia Pulmonar Aguda de Risco Intermediário. A Combinação de Biomarcadores é a Solução?
Source: Arq Bras Cardiol. 2024 Apr 9;121(2):e20240075. [Article in Portuguese] doi: 10.36660/abc.20240075 (PMC11081194; doi:10.36660/abc.20240075)
Supplement: Translation

# Translation: Focus on Intermediate-Risk Acute Pulmonary Embolism. Is the Combination of Biomarkers the Solution?

**Authors:** Carlos Henrique Miranda

**Affiliations:** 1 Ribeirão Preto SP Brazil Divisão de Medicina de Emergência, Departamento de Clínica Médica, Faculdade de Medicina de Ribeirão Preto, Universidade de São Paulo, Ribeirão Preto, SP – Brazil

**Keywords:** Pulmonary Embolism, Biomarkers, Echocardiography

The incidence of acute pulmonary embolism (APE) has been increasing over time throughout the world, including in Brazil. This behavior is probably secondary to the aging of the population associated with increased prevalence and better prognosis related to cancer.^1^

The clinical presentation of APE has two extremes that are easy to identify. A high-risk group, around 4% of patients, which is characterized by circulatory shock or cardiorespiratory arrest, with a high mortality rate. In this group, treatment with thrombolytics is well-established.^2^

At the other extreme, there is the low-risk group, around 40% of patients, which is characterized by a Pulmonary Embolism Severity Index (PESI) score ≤ 2 and the absence of right ventricular (RV) dilation assessed by echocardiography or computed tomography pulmonary angiography (CTPA), with a reduced mortality rate. In this group, anticoagulants, such as enoxaparin or direct-acting oral anticoagulants (DOACs), are sufficient. Early hospital discharge or even outpatient treatment can be considered for these patients.^2^

However, the greatest challenge in clinical practice is the management of intermediate-risk patients, who are between these two extremes. This profile corresponds to most patients with APE, observed in approximately 56% of them. This group is highly heterogeneous, and a percentage of them have a high probability of clinical deterioration, behaving more closely to high-risk patients.^3^

Several biomarkers and imaging tests have been used to improve risk stratification in this intermediate group. Troponin I or T is the most used biomarker for this purpose. The 2019 European Society of Cardiology (ESC) guideline recommends using troponin within a decision-making algorithm.^4^Patients with an elevation of this biomarker associated with RV dilation are reclassified as intermediate-high risk. However, this recommendation was based on expert opinions. Most of the studies did not use ultrasensitive troponin assays, and its cut-off point was not standardized.^5^

B-type natriuretic peptide (BNP) and the N-terminal fraction of pro-BNP (NT-proBNP) can also be used in this setting. A meta-analysis of five more recent studies showed that an NT-proBNP greater than 1000 ρg/ml increased the risk of clinical deterioration. However, this marker was not evaluated within a decision-making algorithm.^6^

Plasma lactate, widely used in patients with sepsis, also showed good predictive accuracy in patients with APE to determine the short term prognosis. Adding lactate measurement to the ESC-2019 algorithm, patients classified as an intermediate-high risk with venous lactate ≥ 3.3 mmol/L had a prevalence of adverse events of 27.5% compared to 6.8% in those with lactate < 3.3 mmol/L.^7^

RV dilation on CTPA assessed through the ratio of RV diastolic diameter to left ventricular diastolic diameter (cut-off value of 0.9 or 1.0) is a prognostic marker, as well as RV dilation, hypokinesia of the RV free wall, and the presence of pulmonary hypertension on echocardiography.^8^

Within this context, Gunes et al.^9^showed in their study that the soluble form of an interleukin-33 receptor called sST2 had good accuracy (79.8%) in predicting the occurrence of death within six months and recurrent hospitalization in patients with APE. These investigators included a convenience sample of 100 patients admitted to their emergency department. Like the vast majority of other studies that evaluated biomarkers in EPA, they studied the performance of this biomarker independently of the initial prognostic classification of these patients. The validation of these biomarkers would need to be performed within a selected sample of intermediate-risk patients, excluding low and high-risk patients. The greatest challenge in clinical practice is precisely defining the prognosis in intermediate-risk patients.

Despite several investigations showing that these markers are independent predictors of worse clinical outcomes, their isolated prognosis predictive performance was unsatisfactory. All these biomarkers showed a low positive predictive value.^6^

The current recommendation is to use these biomarkers within predictive algorithms, considering several variables together. For example, scores derived from prospective studies have been emerging, and they associate several variables, such as the BOVA score.^10^ Figure 1 Patients with a BOVA score > 4 points had a high cumulative incidence of APE-related complications (19.9%). Another score, TELOS, encompasses RV dilation, troponin, and lactate.^11^In a clinical study, the BOVA and TELOS score classified the same proportion of patients in the intermediate-high risk category (5.9% and 5.7%) and with a similar adverse event rate (18.6% and 21.1%), while the ESC 2019 algorithm classified a higher percentage of patients in this category (12.5%; p<0.001) with a lower event rate (13%; p=0.18).^12^

**Figure 1:** Algorithm suggested for acute pulmonary embolism points risk stratification. PESI: Pulmonary Embolism Severity Index; RV: right ventricle; SBP: systolic blood pressure; *defined as echocardiographic assessment RV/LV>0.9, systolic pulmonary artery pressure > 30 mmHg, RV end-diastolic diameter>30mm, RV dilation or RV free-wall hypokinesis or RV/LV>1.0 on CTPA.^†^RV dilatation (end-diastolic diameter > 30 mm) or RV/LV end-diastolic diameter ≥ 1; pulmonary hypertension > 30 mmHg, hypokinesis of the RV free wall;^‡^heart rate/ systolic blood pressure (the value is included in a model);^§^PO2/FiO2 ratio (the value is included in a model);^//^cumulative presence of elevated troponin, elevated NT-proBNP and RV/LV≥1.0.

So far, Gunes et al.^9^have completed the first step of validating the sST2 in APE risk stratification. It is necessary to evaluate the predictive performance of this biomarker in larger and multicenter samples, including essentially intermediate-risk patients, and integrate it within predictive algorithms with other variables.

Combining several variables, including different biomarkers, is probably the best strategy to improve the prognostic stratification in intermediate-risk APE.
